# Antibacterial activity of Hungarian varietal honeys against respiratory pathogens as a function of storage time

**DOI:** 10.1038/s41598-024-60961-3

**Published:** 2024-05-03

**Authors:** Lilla Nagy-Radványi, Viktória L. Balázs, Béla Kocsis, Eszter Csikós, Virág D. Ángyán, Péter Szabó, Viktória Biró, Marianna Kocsis, Ágnes Farkas

**Affiliations:** 1 Department of Pharmacognosy, Faculty of Pharmacy, University of Pécs, 7624 Pécs, Hungary; 2 Department of Medical Microbiology and Immunology, Faculty of Medicine, University of Pécs, 7624 Pécs, Hungary; 3 Institute of Geography and Earth Sciences, Faculty of Sciences, University of Pécs, 7624 Pécs, Hungary; 4 Department of Agricultural Biology, Institute of Biology, University of Pécs, 7624 Pécs, Hungary

**Keywords:** Microbiology, Plant sciences, Health care

## Abstract

Today, antibiotic therapies that previously worked well against certain bacteria due to their natural sensitivity, are becoming less effective. Honey has been proven to inhibit the biofilm formation of some respiratory bacteria, however few data are available on how the storage time affects the antibacterial effect. The activity of black locust, goldenrod, linden and sunflower honeys from three consecutive years (2020, 2021, 2022) was analyzed in 2022 against Gram-negative (*Haemophilus influenzae*, *H. parainfluenzae*, *Pseudomonas aeruginosa*) and Gram-positive (*Streptococcus pneumoniae*) bacteria using in vitro microbiological methods. After determining the physicochemical parameters of honey, broth microdilution was applied to determine the minimum inhibitory concentration of each honey type against each bacterium, and crystal violet assay was used to test their antibiofilm effect. The possible mechanism of action was explored with membrane degradation test, while structural changes were illustrated with scanning electron microscopy. Honeys stored for one or two years were darker than fresh honeys, while older honeys had significantly lower antibacterial activity. The most remarkable inhibitory effect was exerted by linden and sunflower honeys, and *P. aeruginosa* proved to be the most resistant bacterium. Based on our results, honey intended for medicinal purposes should be used as fresh as possible during a treatment.

## Introduction

Respiratory infections are extremely common, being one of the main causes of illness and death worldwide, and one of the most significant problems in clinical medicine. In the beginning, the symptoms are caused by viruses, and this is often followed by bacterial superinfection, primary bacterial infection develops only in a smaller proportion^1^. Regarding the resistance conditions of the most important upper respiratory tract pathogens, it can generally be said that macrolide and trimethoprim/sulfamethoxazole resistance is significant, and the number of penicillin-resistant or moderately sensitive bacterial strains is increasing. Antibiotic resistance can also occur in natural populations to a small extent, however, the clinical use and overuse of certain antibiotics exerts a strong selection pressure on the emergence of resistance^2–4^.

Bacterial communities colonize both the upper and lower respiratory tracts, but the highest bacterial density is found in the upper respiratory tract (URT). A characteristic of respiratory infections is that the causes of inflammation are actually members of the naturally present bacterial flora of the respiratory tract and primarily cause disease in an immunosuppressed state. Potentially infectious genera such as *Streptococcus* spp., *Haemophilus* spp., *Pseudomonas* spp. or *Moraxella* spp. can also be found in the microbiome of a healthy person^5,6^. These microbial communities often form biofilms, and mature biofilms show tolerance to environmental stressors, antibiotics and the body's immune responses. The bacterial biofilm provides optimal conditions and protection for the entire bacterial community^7,8^. The high degree of resistance can be attributed to the metabolic changes of the cells connected in the biofilm and the structural characteristics affecting the permeability of the drugs^9^. Extracellular polysaccharides (EPS), which play an important role in the formation of biofilms, can prevent the antibiotic from reaching the bacterial DNA, thereby providing a high degree of resistance to bacterial colonies^10,11^. Bacteria embedded in the polysaccharide matrix communicate with each other, the metabolism takes place through the channels of the matrix^12^. With the help of horizontal gene transfer, bacterial cells are able to transfer antibiotic resistance genes to each other, hence it is not necessary to develop resistance to a given agent from generation to generation^13^. In order to ensure the effectiveness of the treatment, it is definitely necessary to reduce the formation of biofilm or to destroy the biofilm, because 80% of bacterial infections are due to biofilm formation^14^.

*Pseudomonas aeruginosa* and *Streptococcus pneumoniae*, as well as *Haemophilus* species, are among the most frequently isolated respiratory bacteria capable of forming biofilms^15^. *P. aeruginosa* is a particularly dangerous Gram-negative opportunistic pathogenic bacterium, as most of its strains are antibiotic resistant^16^. The biofilm layer it creates reduces the surface area suitable for breathing in the lungs, thus creating optimal conditions for the reproduction of other microbes^17^. Two members of the Gram-negative *Haemophilus* genus, *H. influenzae* and *H. parainfluenzae*, are frequent colonizers of the respiratory system and can cause local and systemic infections and pose a serious threat to the human body. Due to the adhesion of biofilms, patients are exposed to increased risk, as antibiotic therapy is not successful in some cases^18,19^. In contrast to the pathogens mentioned so far, *S. pneumoniae* is a Gram-positive bacterium, and the symptoms of the disease are rather due to the inflammatory reactions than to the invasiveness of the microbe. It is a capsulated bacterium, which makes it easy to stick to the surface of the respiratory tract^20^. During pneumococcal infections, the bacterium adheres to the surface of the bronchi and creates a biofilm, thereby making the treatment of the infection more difficult. In many cases, biofilm structures can also be observed in certain areas of the lungs^21,22^. In light of these facts, and as a result of the large increase in antibiotic resistance, the use of natural agents and a more thorough knowledge of the microorganisms that cause diseases affecting the respiratory system become even more relevant^23^.

The antibiofilm effect of honey has been confirmed in several clinical studies, so its use as an additional therapy can be significant in the case of respiratory tract infections^24^. The glucose oxidase enzyme present in honey is secreted by the pharyngeal gland of the bee and its task is to convert glucose into gluconic acid δ-lactone, producing hydrogen peroxide as a by-product. The fact that the H_2_O_2_ content of honey is essential for its antibacterial activity has been supported by several research groups, with the maximum H_2_O_2_ concentration observed at honey dilutions between 15 and 50% (w/v)^25^, except for manuka honey, where H_2_O_2_ does not accumulate^26^. The hydrogen peroxide responsible for the bactericidal effect breaks down into water and oxygen over time, so its level decreases thanks to the plant-derived catalase enzyme. Based on these, it can be assumed that honeys stored for years lose their antibacterial effect due to the decrease in the activity of the enzyme glucose oxidase^27,28^. H_2_O_2_ can also be produced as a result of the prooxidant action of the biologically active polyphenols present in large quantities in honey. In this case, the pH value is an important parameter, it determines whether these compounds act as antioxidants or have an antibacterial effect^29,30^. However, low pH and high sugar content are often sufficient to inhibit microbial growth^31^.

During the antibiofilm activity, the active ingredients of honey diffuse into the biofilm matrix and, in addition to destroying the biofilm, prevent further biofilm formation by inhibiting adhesion processes^32^. Proano et al. observed that osmotic activity, H_2_O_2_ content, high sugar concentration and the presence of defensin-1 peptide are responsible for the hypertension in honey, and this condition leads to the suppression of biofilm formation^33^. H_2_O_2_ disrupts the attachment of bacterial cells and destabilizes the biofilm matrix, while bee-derived defensin-1 inhibits the formation of extracellular polymeric substances in addition to initial adhesion^34,35^. Polyphenolic components also prevent bacterial cells from connecting to each other or attaching to a given surface (biotic, abiotic), thus disrupting one of the most sensitive steps of biofilm formation. In addition to all this, polyphenolic compounds can inhibit the synthesis of EPS^36^. Sindi and his research team (2019) found that honey can reduce the number of viable bacterial cells within the mixed biofilm, and honey treatment also has a significant effect on metabolic activity^37^.

For the therapeutic use of honey, it is essential to ensure its quality. Honey can be contaminated with various microorganisms, including endospores of *Clostridium botulinum* and *C. tetani*. This fact is often overlooked, and in order to avoid a possible infection, it is important to sterilize honey intended for medicinal purposes^38^. In addition, nectar-producing plants are often sprayed with various herbicides and pesticides, compounds that also appear in honey. Environmental pollution, heavy metals from industrial areas, and antibiotic treatment of bees can also affect the quality of honey. Because of this, a clear definition of "medical grade honey" is definitely necessary to guarantee the safety and effectiveness of honeys during a treatment. Medical grade honey must fulfil the following criteria: “(1) organic, free of contaminants and toxic substances; (2) gamma-sterilized under standardized conditions, free of dangerous microorganisms; (3) can be safely implemented in medical therapies; (4) follows strict production and storage standards, legal and safety regulations; (5) complies to physicochemical criteria that are important for the use of honey as a wound care product”^39^.

The amount of catalase enzyme, the composition of the active ingredients and, in this context, the complex effect of different types of honeys can show significant differences depending on which species or region the honey comes from. The basic question of our research is to what extent storage time, and the botanical and geographical origin of four Hungarian varieties of honey (black locust, goldenrod, linden, sunflower) influence their antibacterial effect, and against which biofilm-forming bacterial strains is the antibacterial effect of each honey sample most effective. To our knowledge, no similar comparative study covering several years has been carried out yet for Hungarian varietal honeys.

## Methods

### Honey samples

For our tests, we used black locust/acacia (*Robinia pseudo-acacia*), goldenrod (*Solidago gigantea*), linden (*Tilia* spp.) and sunflower (*Helianthus annuus*) honeys purchased from the same Hungarian apiaries in three consecutive years (2020, 2021, 2022). The examination of the physicochemical parameters of honey and the testing of its antibacterial effect took place in 2022, not only in 2022 samples, but also in the case of samples from 2020 and 2021. The honey samples came from the southern Transdanubian region and were stored at room temperature (20–21 °C) and in the dark until used.

### Melissopalynological analysis

In order to establish the true type of honeys, it is crucial to clarify their exact botanical origin, so we performed a melissopalynological analysis following the method of Von der Ohe et al.^40^. Ten grams of our honey samples were added to centrifuge tubes and mixed with 20 mL of distilled water using a Combi-spin FVL-2400N Vortex (Biocenter Kft., Szeged, Hungary). The subsequent centrifugation lasted for 10 min at a speed of 8753×*g* with a Neofuge 15R centrifuge (Lab-Ex Ltd., Budapest, Hungary), after which the supernatant was poured off. This step was followed by another centrifugation (5 min, 8753×*g*) and decantation, then 10 mL of distilled water was measured to the sediment left from the first centrifugation. In what follows, 250 μl of distilled water was added to the sediment remaining in the centrifuge tube, and after vortexing, 20 μl of the thus prepared pollen suspension was pipetted onto a microscope slide which was then placed on a heating plate set to 40 °C (OTS 40, Tiba Kft., Győr, Hungary). The water was evaporated, and then a small piece of Kaiser’s glycerin jelly with fuchsine (Merck Life Science Ltd., Budapest, Hungary) was added to each pollen sample. When the jelly melted, the preparations were covered with a coverslip. Pollen preparations were analyzed with Nikon Eclipse E200 microscope equipped with a Michrome 20MP CMOS digital camera (Auro-Science Consulting Kft., Budapest, Hungary), and photomicrographs were taken at 400 × magnification using 4.3.0.605 version of TCapture software^41^. At least 500 pollen grains were counted per honey sample, indicating how many pollen grains belong to a given plant species, genus or family. The relative frequency of pollen types was given as a percentage of all pollen grains.

### Physicochemical parameters

The color intensity of the varietal honeys used in the tests was determined according to Beretta et al.^42^. The 50% (w/w) honey solutions prepared were placed in an ultrasonic water bath (water temperature: 45–50 °C) for 5 min and then filtered (0.45 µm pore size, Agilent Technologies, Milan, Italy). Color intensity results from the difference in absorbance values measured at 450 and 720 nm using a Shimadzu UV-1800 spectrophotometer (Shimadzu Schweiz GmbH, Reinach, Switzerland) were expressed in milliabsorbance units (mAU).

A DSZ-708 Multiparameter analyzer (Simex Ltd., Budapest, Hungary) was used to examine pH values and electrical conductivity. The pH value of the honey samples was determined according to the Codex Alimentarius and the pH meter was calibrated each time with buffer solutions at values of 4 and 9^43^. For the measurement, 10 g of honey was dissolved in 75 ml of freshly distilled carbon dioxide-free water, and in the case of electrical conductivity, a 20% honey solution was prepared with distilled water. The electrical conductivity of honey means the conductivity of the honey solution measured at 20 °C, the results were expressed in milli-Siemens per centimeter (mS/cm)^44^.

### Culture bacteria

The antibacterial effect of varietal honeys was investigated in case of both Gram-negative and Gram-positive bacteria. *Haemophilus influenzae* (DSM 4690) and *H. parainfluenzae* (DSM 8978) strains were grown in a supplemented Mueller Hinton broth special. 500 µl *Haemophilus* supplement B (Diagon Kft., Budapest, Hungary) and 750 µl (1 mg/ml) NAD were added to 3750 µl of Mueller–Hinton II Broth (MHB, Oxoid Ltd., London, UK). For *Pseudomonas aeruginosa* (ATCC 27853) and *Streptococcus pneumoniae* (DSM 20566), 100 ml of sterile MHB medium was used. The incubation time of bacterial suspensions was 12 h at 37 °C in a shaker incubator at 60 rpm (C25 Incubator Shaker, New Brunswick Scientific, Edison, NJ, USA)^45^. Before the tests, the bacteria were diluted to the given concentration with the appropriate medium.

### Determination of minimum inhibitory concentrations (MIC) and minimum bactericidal concentration (MBC)

A broth microdilution test was used to determine the minimum inhibitory concentrations (MIC), which is commonly used in microbiological laboratories in accordance with CLSI guidelines (Clinical & Laboratory Standards Institute)^46^. The procedure was performed on 96-cell microtiter plates. Three dilution series were used, as we expected—based on preliminary experiments—that the antibacterial activity would decrease with increasing storage time. The following dilution series were prepared (using Mueller–Hinton broth): 42.5, 45, 47.5, 50, 52.5, 55, 57.5% (w/w) (2020 honeys), 25, 27.5, 30, 32.5, 35, 37.5, 40% (w/w) (2021 honeys) and 10, 12.5, 15, 17.5, 20, 22.5, 25% (w/w) (2022 honeys). Then 100 µL of the honey solutions and bacterial suspensions (10^5^ CFU/mL) were measured into one cell of the microtiter plate, followed by incubation at 37 ℃ for 24 h. We performed our tests with six repetitions and considered the lowest honey concentration as the MIC, in which case there was no visible bacterial growth in the cells.

After the MIC determination, 10 µL of culture medium was taken from each cell of the microtiter plate, where no visible growth was observed, to test the minimum bactericidal concentration (MBC). In this case, the samples were cultured on BA plates and the incubation at 37 °C lasted for 24 h. Finally, we read the lowest concentration where no bacterial growth was observed on the plates^47^.

### Biofilm inhibition study

The biofilms were formed on 96-cell microtiter plates applying the crystal violet (CV) assay, thereby testing the anti-biofilm effect of the honey samples^48^. The treatments were performed with honey samples with a concentration of MIC/2 and a bacterial suspension nutrient solution with a cell count of 10^8^ CFU/mL was used as a positive control, and a cell-free honey nutrient solution was used as a negative control. As a first step, 200 μL of bacterial suspension was measured into one cell of the microtiter plate and the subsequent incubation at 37 °C lasted for four hours, thereby promoting the adhesion of the bacterial cells. The non-adherent cells were removed with physiological saline and only then were the honey solutions pipetted into the cells. In this case, the incubation was 24 h at 37 °C, and then the non-adherent cells were washed again with physiological saline. In order to fix the cells, 200 μL of methanol was added to each well for 15 min at room temperature (RT). In order to stain the bacterial biofilm, 200 μL of 0.1% crystal violet dye was applied after removing the methanol. After 20 min (RT), the excess dye was removed with water, and the crystal violet bound to the biofilm was dissolved with 200 μL of 33% acetic acid per cell. The absorbance values were determined at 590 nm using a plate reader (BMG Labtech SPECTROstar Nano, Budapest, Hungary). Among other things, the crystal violet dye binds to extracellular polysaccharides (EPS) which play an important role in the formation of biofilms, therefore enabling the estimation of the total biomass of the biofilm in the cell of the microtiter plate. The inhibition rate was determined based on the following formula: (1 − S/C) × 100% (C and S were defined as the average absorbance of control and sample groups, respectively)^49^.

### Membrane degradation assay

During the study of cell material release, each bacterial suspension (10^8^ CFU/mL) was prepared in PBS (phosphate buffer saline) and bacterial cells without honey treatment were used as a control. The bacterial cells were treated with honey samples of 20, 40 and 60% (w/w) concentration for one hour. In addition, the time dependence of membrane degradation was also investigated, in this test the bacterial cells were suspended in PBS containing 60% (w/w) honey. The treatments lasted: 0, 10, 20, 40, 60 and 90 min. After the treatments, the bacterial cells were centrifuged in both cases (Neofuge 15R, Lab-Ex Ltd., Budapest, Hungary) at 12,000×*g* for 2 min. The absorbance of the supernatant containing nucleic acid was measured at 260 nm with a Metertech SP-8001 (Abl&e-Jasco Ltd., Budapest, Hungary) spectrophotometer. The results were expressed as a percentage, compared to the control^50^.

### Scanning electron microscopy (SEM)

In order to visualize the inhibitory effect of honey on biofilm and to illustrate the structural changes, SEM images were taken. Both Gram-positive (*S. pneumoniae*) and Gram-negative (*P. aeruginosa*) bacteria were included in the study, and the samples were treated with linden honey which has an effective antibiofilm effect.

During the procedure, biofilms were formed on degreased and sterilized coverslips after the plates were incubated for 4 h at 37 °C in 5 mL of bacterial suspension (10^5^ CFU/mL). After the adhesion, washing with physiological saline followed, and then linden honey from three different years was applied at a concentration of MIC/2 (5 mL). Untreated coverslips were used as controls. After 24 h of incubation (37 °C), non-adherent cells were washed again, and this was followed by 2 h (RT) incubation in 2.5% glutaraldehyde to fix the biofilm. The samples were dehydrated in absolute ethanol (50%, 70%, 80% and 90% solutions) for 2 × 15 min (RT). As a next step, the coverslips were placed in a 1:2, 1:1, 2:1 mixture of t-butyl alcohol and absolute ethanol. The samples were then transferred to absolute t-butyl alcohol for 1–1 h (RT) followed by overnight freeze-drying. The examination of biofilms coated with a gold membrane was performed with a JEOL JSM IT500-HR scanning electron microscope (Jeol Ltd., Tokyo, Japan)^51^.

### Statistical analysis

**Figure 1:**
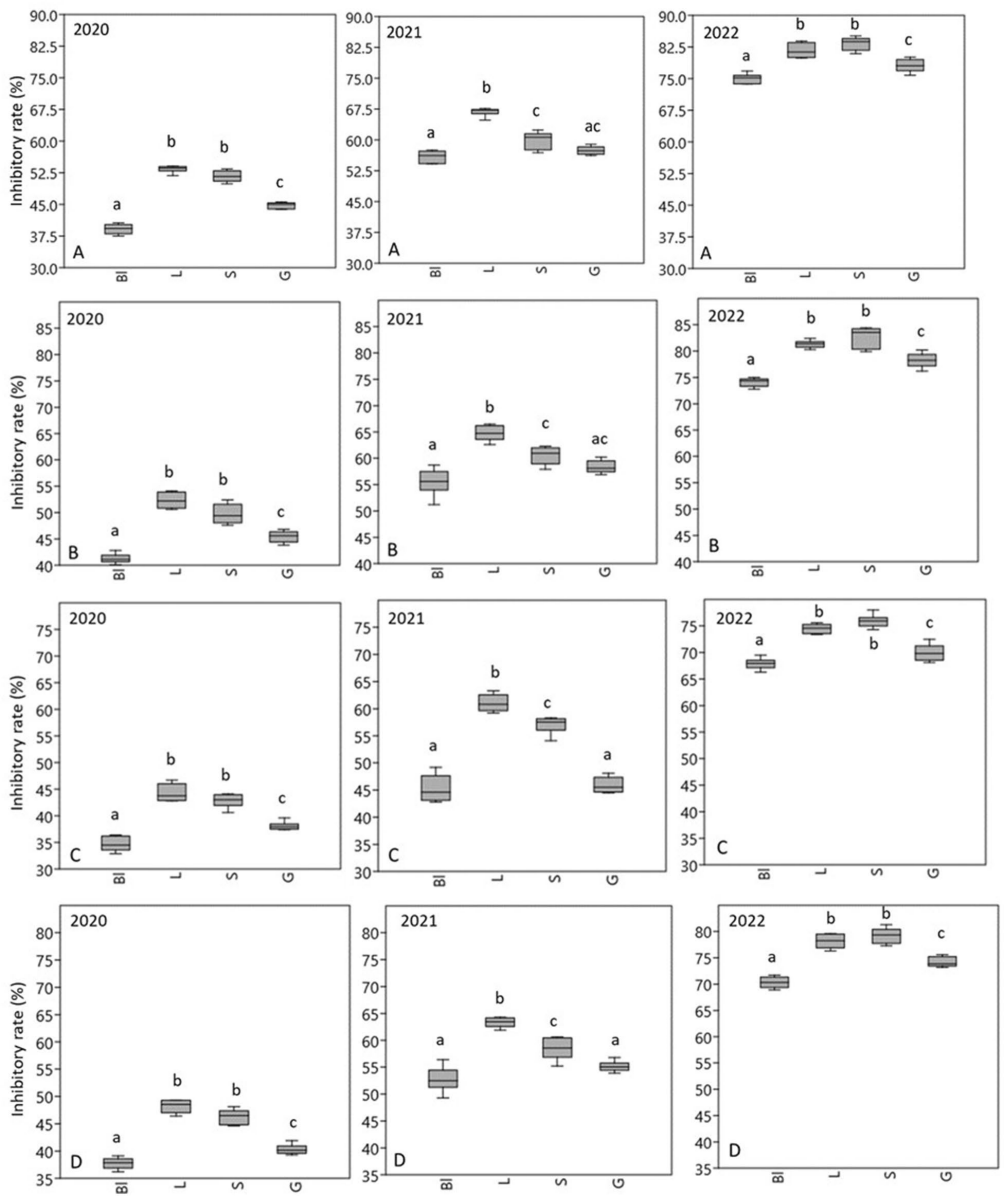
Biofilm inhibitory rates of honey samples against *H. influenzae* (**A**), *H. parainfluenzae* (**B**), *P. aeruginosa* (**C**) *S. pneumoniae* (**D**). Bl-black locust honey, L-linden honey, S-sunflower honey, G-goldenrod honey. Data are expressed using box plots, minimum to maximum values are presented by vertical lines, while median within the plot as horizontal line. Lowercase letters above the boxes indicate significant differences between the means of inhibitory rates, the same lowercase letters above the boxes indicate mean values that are not significantly different from each other; different lowercase letters indicate means, which are significantly different (*p* ≤ 0.01).

Statistical analyses were carried out using Excel® (Microsoft Corp., Redmond, WA, USA) and the PAST software package version 3.11^52^ after normality checking with the Shapiro–Wilk test. Data were expressed as means ± standard deviations (SD) in case of honey type absorbance (Table 2) and membrane degradation results (Tables 5, 6). Results of antibiofilm activity (Fig. 1) were expressed as median (minimum to maximum) in box plots. The honey types had been compared with each other based on a given parameter using one-way ANOVA. If the null hypothesis of the ANOVA was rejected, we used Student t-test to establish a difference between the two group pairs (honey types). The p-values at 1% (p ≤ 0.01) or 5% (p ≤ 0.05) were considered significant.

## Results

### Melissopalynological analysis and Physicochemical parameters of honey samples

Based on the pollen analysis and examination of the physicochemical properties of the honeys included in the research, all honey samples can be considered unequivocally as varietal honey (Tables 1 and 2). In black locust honeys, *R. pseudoacacia* pollen was the dominant pollen type in accordance with the honey type declared by the beekeeper, similarly to goldenrod, linden and sunflower honeys which also contained high amounts of *Solidago*, *Tilia* and *Helianthus* pollen, respectively.

**Table 1:** Relative frequency of pollen types in the studied honeys.

Honey samples		Pollen type − relative frequency (%)						
		*Robinia*	*Solidago*	*Helianthus*	*Tilia*	*Brassica*	Asteraceae	Other
Black locust	2020	56.1	2.4	3.8	16.2	13.1	3.6	4.8
	2021	51.8	-	8.8	14.5	8.3	9.1	7.5
	2022	46.6	3.7	6.5	10.3	20.6	1.9	10.4
Goldenrod	2020	1.7	63.2	2.3	0.6	7.6	9.8	14.8
	2021	4.8	73.4	3.6	1.9	6.7	8.4	1.2
	2022	0.8	60.6	6.3	2.6	10.2	7.8	11.7
Linden	2020	18.8	0.7	16.3	47.2	1.7	4.4	10.9
	2021	20.3	1.5	10.2	53.6	2.1	5.6	6.7
	2022	22.6	2.2	11.2	45.5	3.2	5.9	9.4
Sunflower	2020	13.2	3.4	66.5	8.4	–	3.6	4.9
	2021	16.4	2.7	58.7	13.8	0.5	1.8	6.1
	2022	5.5	3.2	69.6	4.2	–	7.6	9.9

Dominant pollen > 45%, secondary pollen 16–45%, important minor pollen 3–15%, minor pollen < 3% of the pollengrains counted.

**Table 2:** Sensory characteristics and physicochemical parameters of honey samples.

Honey type, plant name	Year	Sensory characteristics (color, odor and consistency)	ABS_450–720_ (mAU)	Electrical conductivity (mS/cm)	pH
Black locust, *Robinia pseudoacacia*	2020	Light amber, weak odor, liquid, viscous	103.1 ± 3.0^a^	0.132 ± 0.01	3.30 ± 0.02
	2021	Light amber, weak odor, liquid, viscous	99.3 ± 3.3^a^	0.121 ± 0.01	3.22 ± 0.03
	2022	Pale, yellowish green, weak odor, liquid, viscous	44.4 ± 1.7^b^	0.126 ± 0.01	3.27 ± 0.06
Goldenrod, *Solidago gigantea*	2020	Dark amber, moderately intense odor, semisolid, fine granulated	280.7 ± 1.5^a^	0.624 ± 0.01	3.56 ± 0.03
	2021	Dark amber, moderately intense odor, semisolid, fine granulated	279.4 ± 1.9^a^	0.609 ± 0.01	3.52 ± 0.04
	2022	Amber, moderately intense odor, semisolid, fine granulated	236.9 ± 3.2^b^	0.615 ± 0.00	3.59 ± 0.03
Linden, *Tilia* spp.	2020	Amber, strong odor, semisolid fine granulated	221.9 ± 1.7^a^	0.567 ± 0.03	4.27 ± 0.04
	2021	Amber, strong odor, semisolid fine granulated	211.1 ± 3.4^b^	0.607 ± 0.02	4.32 ± 0.03
	2022	Light amber, strong odor, semisolid, fine granulated	166.3 ± 4.0^c^	0.592 ± 0.02	4.19 ± 0.03
Sunflower, *Helianthus annuus*	2020	Dark golden yellow, weak odor, semisolid, coarse granulated	422.8 ± 2.0^a^	0.221 ± 0.01	3.68 ± 0.04
	2021	Dark golden yellow, weak odor, semisolid, coarse granulated	413.5 ± 4.2^a^	0.224 ± 0.01	3.61 ± 0.05
	2022	Golden yellow, weak odor, semisolid, coarse granulated	116.6 ± 1.5^b^	0.223 ± 0.01	3.66 ± 0.04

ABS_450–720_: absorbance of diluted honey samples referring to their color. Data are means ± standard deviations of three independent determinations (n = 3). Different lowercase letters indicate a significant difference among years according to Student's *t*-test (*p* ≤ 0.05).

The color of honey is one of the most variable parameters, which can range from white, through pale yellow and red, to black. In this case, the determining factor is the plant origin and the storage time. The color of fresh black locust honey was pale, yellowish green, but the stored samples were darker (44.4 ± 1.7 vs. 103.1 ± 3.0 mAU), approaching the color of light amber colored linden honey. We observed a similar color change in the case of the other honey samples. The most obvious contrast between fresh and stored honey colors was experienced in sunflower honeys (116.6 ± 1.5 vs. 422.8 ± 2.0 mAU). The highest difference in color intensity was measured between honey samples stored for two vs. one year, afterwards we did not observe any significant difference in smell and texture in the next 2 years.

The pH of honey mainly depends on dissociated acids, which affects both the development of microorganisms and enzyme activity. The pH value of the varietal honeys varied between 3.22 and 4.32. Regarding the average of 3 years, the highest values were measured in linden honeys (4.26 ± 0.07), the lowest values in black locust honeys (3.26 ± 0.04). Also electrical conductivity was the lowest in black locust honey (0.126 ± 0.01 mS/cm), followed by sunflower (0.222 ± 0.002 mS/cm), linden (0.589 ± 0.02 mS/cm) and goldenrod honey (0.616 ± 7.2 mS/cm). The pH and electrical conductivity did not change with storage time, so similar values were obtained for honeys of the same variety from different years.

### MIC and MBC determination

The MIC and MBC values of the investigated honey samples showed variation in terms of the storage time, the type of honey, and the bacterial strains included in the study (Tables 3 and 4). Based on our results, it can be observed that as the storage time increased, higher concentrations of honey solutions were needed to achieve the appropriate inhibitory effect. In the case of the 2020 honeys, the MIC values ranged between 42.5 and 50%, while for the 2022 samples, between 10 and 17.5%. A difference of a similar magnitude was also experienced for the MBC values.

**Table 3:** MIC values of honey samples.

MIC values (%)		*H. influenzae*	*H. parainfluenzae*	*P. aeruginosa*	*S. pneumoniae*
Black locust	2020	50	50	50	50
	2021	30	30	35	32.5
	2022	12.5	12.5	17.5	12.5
Goldenrod	2020	47.5	47.5	50	50
	2021	27.5	27.5	35	30
	2022	12.5	12.5	17.5	12.5
Linden	2020	42.5	42.5	47.5	45
	2021	25	25	32.5	27.5
	2022	10	10	12.5	10
Sunflower	2020	42.5	42.5	47.5	45
	2021	25	25	32.5	30
	2022	10	10	12.5	10

Percentage values of the table correspond to dilution % of honey causing antimicrobial effects.

**Table 4:** MBC values of honey samples.

MBC values (%)		*H. influenzae*	*H. parainfluenzae*	*P. aeruginosa*	*S. pneumoniae*
Black locust	2020	55	55	57.5	57.5
	2021	37.5	37.5	40	37.5
	2022	20	20	25	20
Goldenrod	2020	55	55	57.5	55
	2021	35	35	40	35
	2022	20	20	25	20
Linden	2020	47.5	47.5	52.5	50
	2021	30	30	35	32.5
	2022	15	15	20	15
Sunflower	2020	50	50	55	50
	2021	32.5	30	37.5	35
	2022	15	15	20	15

Percentage values of the table correspond to dilution % of honey causing antimicrobial effects.

Linden and sunflower honey exerted the most significant inhibitory effect, while black locust honey was the least active. *Haemophilus* strains were the most sensitive, while *P. aeruginosa* proved to be the most resistant bacterium.

### Antibiofilm activity

When examining the inhibitory effect of honey on biofilm formation, the individual honeys were used at a concentration of MIC/2. It is clearly visible also in this series of tests that the antibacterial activity of stored honeys is lower than that of fresh honeys (Fig. 1). When measured in 2022, the average inhibition rate of 2020 honeys (34.7–53.4%) was about half of that of 2022 honeys (67.9–83.2%). The 2021 samples inhibited biofilm formation by 45.3–66.8%. The antibiofilm effect of linden and sunflower honey was the most remarkable, the inhibition rate for the 2022 samples reached 80% against *Haemophilus* strains. Black locust and goldenrod honeys showed lower activity, but the fresh samples from 2022 also inhibited biofilm formation by an average of 70% in the selected bacterial strains. Considering each sampling year, the honey samples were able to exert the most significant antibiofilm effect against *Haemophilus* strains, while their activity was lower in the case of *P. aeruginosa* and *S. pneumoniae* strains.

### Membrane degradation

To demonstrate one of the mechanisms of action of honey, we chose linden honey, which showed high activity in previous studies. In this series of experiments, we also worked with samples from three consecutive years and studied the membrane-degrading effect of honey solutions of different concentrations (20, 40, 60, 90%) in the case of Gram-negative (*P. aeruginosa*) and Gram-positive (*S. pneumoniae*) bacteria (Table 5). In the 2020 linden honey treatment, the loss of integrity of the bacterial membrane was observed at concentrations of 60% and above, while when the 2021 sample was used, membrane degradation occurred already at a concentration of 40%. The highest release of cell material was measured when 2022 linden honey was used, here some activity occurred even at the low concentration of 20%. Using the 2022, 60% honey treatment, the proportion of lysed cells for the *S. pneumoniae* bacterium reached 43.7%.

**Table 5:** Effect of linden honeys on the release of DNA in Gram-negative (*P. aeruginosa*) and Gram-positive (*S. pneumoniae*) bacteria.

Linden honey		DNA release from bacterial cells (%)	
Year	Concentrations (%)	*P. aeruginosa*	*S. pneumoniae*
2020	0	0	0
	20	0	0
	40	0	0
	60	7.6 ± 0.9^a^	9.7 ± 1.3^b^
	90	45.2 ± 2.5^a^	49.9 ± 3.0^b^
2021	0	0	0
	20	0	0
	40	8.7 ± 1.3^a^	11.3 ± 2.1^b^
	60	18.8 ± 2.3^a^	21.6 ± 2.6^a^
	90	56.2 ± 2.5^a^	60.1 ± 2.9^b^
2022	0	0	0
	20	10.9 ± 2.1^a^	15.2 ± 2.4^b^
	40	28.7 ± 2.1^a^	38.6 ± 1.2^b^
	60	39.2 ± 1.7^a^	43.7 ± 2.8^b^
	90	100	100

Data are means ± standard deviations of six independent determinations (n = 6). Different lower case letters in the same row for each year indicate significant differences among the two bacteria according to Student’s *t*-test (*p* ≤ 0.05).

In order to investigate the kinetics of cell material release, the 60% solution of the samples was measured at different time intervals (20, 40, 60, 90 min). This experiment showed how many minutes it took for the bacterial membrane to start breaking down after the treatment with linden honey from different years (Table 6). The release of DNA from the bacterial cells started at 20 and 60 min in case of 2022 and 2020 linden honey, respectively. Based on our results, Gram-negative *P. aeruginosa* proved to be a more resistant bacterium in this case as well (Tables 5 and 6).

**Table 6:** Kinetics of 260 nm absorbing material released from Gram-negative (*P. aeruginosa*) and Gram-positive (*S. pneumoniae*) bacteria treated with 60% (w/w) linden honeys.

Linden honey		DNA release from bacterial cells (%)	
Year	Time (min)	*P. aeruginosa*	*S. pneumoniae*
2020	0	0	0
	20	0	0
	40	0	0
	60	7.6 ± 0.9^a^	9.7 ± 1.3^b^
	90	9.8 ± 1.3^a^	10.5 ± 2.2^a^
2021	0	0	0
	20	0	0
	40	12.4 ± 2.3^a^	14.2 ± 2.3^a^
	60	18.8 ± 2.3^a^	21.6 ± 2.6^a^
	90	23.2 ± 1.8^a^	2.3 ± 3.1^b^
2022	0	0	0
	20	22.5 ± 2.5^a^	26.5 ± 1.8^b^
	40	35.1 ± 2.5^a^	40.5 ± 2.2^b^
	60	39.2 ± 1.7^a^	43.7 ± 2.8^b^
	90	65.1 ± 3.0^a^	69.8 ± 2.2^b^

Data are means ± standard deviations of six independent determinations (n = 6). Different lower case letters in the same row for each year indicate significant differences among the two bacteria according to Student’s *t*-test (*p* ≤ 0.05).

### Scanning electron microscopy (SEM)

**Figure 2:**
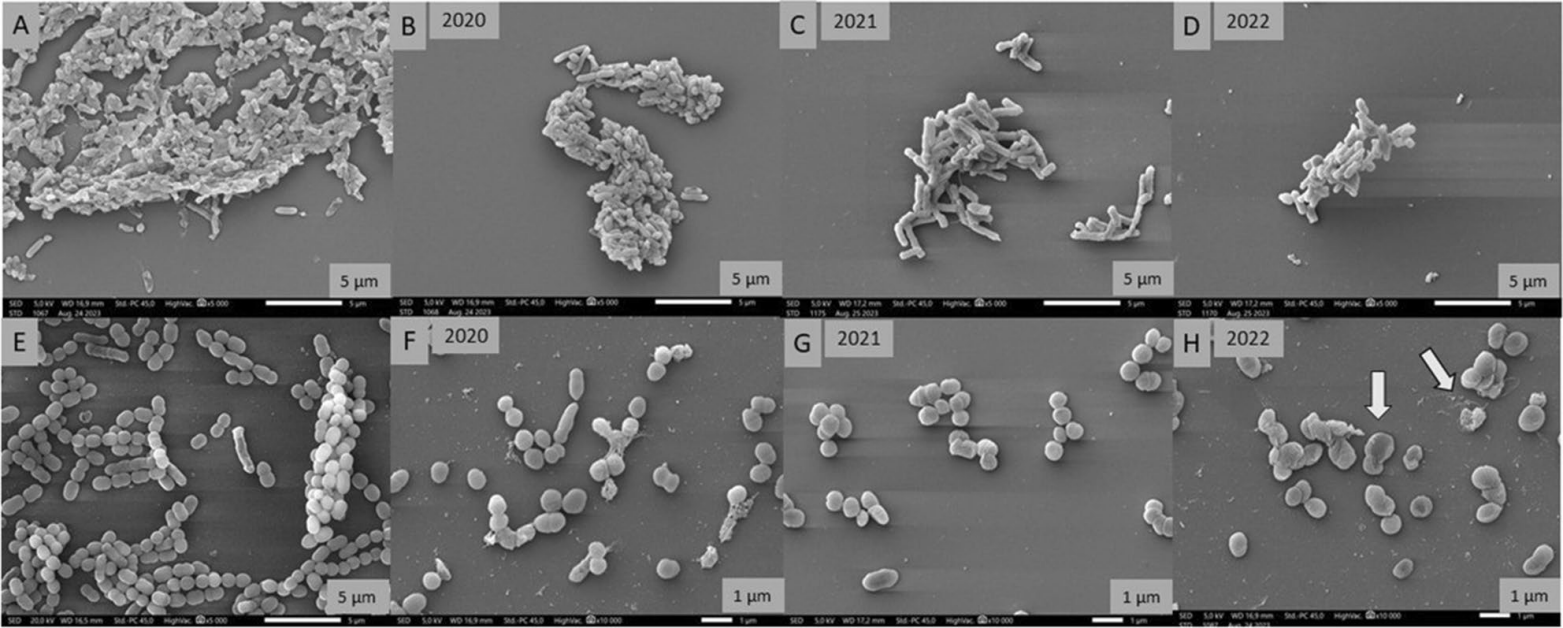
Scanning electron microscopic images of *P. aeruginosa* (**A**–**D**) and *S. pneumoniae* (**E**–**H**). Control samples of bacterial strains (**A**, **E**); treatment with linden honey from different years with MIC/2 concentration in the case of *P. aeruginosa* (**B**–**D**) and *S. pneumoniae* (**F**–**H**)*.*

The inhibitory effect of highly active linden honey on the biofilm formation of Gram-negative (*P. aeruginosa*) and Gram-positive (*S. pneumoniae*) bacteria was illustrated in SEM images (Fig. 2). In the case of untreated samples, the three-dimensional structure of bacterial biofilms was formed (Fig. 2A,E), while in the case of treated samples, biofilm formation was inhibited to an extent corresponding to the age of the honeys. The 2020 and 2021 linden honey reduced biofilm formation to a lesser extent (Fig. 2B,C,F,G) than the 2022 (Fig. 2D,H), similar to previous results. *S. pneumoniae* bacterial cells treated with 2022 linden honey were crushed and their cell material burst out (Fig. 2H). In addition, it can be observed in the images of the untreated samples that *P. aeruginosa* produces a larger amount of biofilm than *S. pneumoniae* during the same time, thus contributing to the increase of its resistance (Fig. 2A,E).

## Discussion

Prior to our current research, we had already demonstrated the antibiofilm effect of several Hungarian varieties of honey against bacterial strains that cause respiratory and wound infections^53,54^, however, no studies have yet been conducted to determine the extent to which honeys stored for several years lose from their antibacterial activity. We once again included respiratory biofilm-forming bacteria in our series of experiments, as there are still few data available on the reaction of these bacteria after treatment with honey, and respiratory diseases of bacterial origin are a serious problem worldwide. Our research work, which has been going on for several years, helps us to get to know these bacteria more thoroughly, as well as to develop alternative complementary therapies that could reduce the risk of developing antibiotic resistance. In the present study, we demonstrated for the first time how the antibiofilm effect of stored vs. fresh honey samples changes in case of four different types of Hungarian honeys against both Gram-positive and Gram-negative respiratory bacteria. In addition, we were able to gain insight into the kinetics and possible mechanism of the antibacterial effect with the help of membrane degradation tests. As a limitation of our study, it has to be noted that not the same honey samples were studied in three consecutive years, but the same honey type harvested in the same geographical region in three consecutive years, with highly similar pollen profile each year.

Based on the examination of the physicochemical parameters of the samples, the electrical conductivity, which is closely related to the pH and macro- and microelement content, proved to be constant over the years, and several research groups measured values similar to our results^55–58^. However, the color intensity of stored honeys was higher, reflecting their darker color compared to fresh honeys. Brudzynski and Kim^59^ measured a lower degree of color change for lighter honeys (e.g. borage) than for darker honeys (e.g. sunflower). We also observed a significant difference in the color of stored vs. fresh sunflower honey, but the difference was also relevant for the lighter black locust honey. The change was not so remarkable in the lighter linden honey and the darker goldenrod honey, so it is assumed that the type of honey and not the starting color of the honey is the decisive factor in the degree of change in color intensity.

Several research groups found that the antibacterial effect of most types of honey is largely dependent on the H_2_O_2_ content^60,61^, but low pH, high sugar content (osmolarity), and the presence of antimicrobial proteins/peptides also all contribute to the antibacterial activity of the honey^31,62^. In general, the higher the hydrogen peroxide and total polyphenol content of a given honey is, the more significant its bacteriostatic and bactericidal effects are^60^. The advantage of the non-peroxide antibacterial effect is that it remains intact even after a longer storage time^63^, while H_2_O_2_ degrades over time thanks to the catalase enzyme^28^. From this, we can conclude that H_2_O_2_ plays a key role in the process of changing activity, since the ratio of the other components responsible for antibacterial activity (non-peroxides) does not change to such an extent over the years^63^. However, high heat and light exposure can also contribute to the degradation of biologically active polyphenols^64^.

Our research has proven that even honeys stored for one year display lower levels of antibacterial effect, and in the case of two-year-old samples, lower activity was even more pronounced. The antibiofilm activity of the 2021 honey samples was lower by an average of 20–25%, however, the inhibition ratio of the linden honeys was over 60% against all bacterial strains even after one year. Our results suggest that the strong antibacterial effect of linden honey is partially attributable to the non-peroxide factor(s), as reported by Farkasovska et al.^61^. Compared to the 2022 harvest, the antibiofilm effect of the 2020 honeys was almost halved. During the study of buckwheat honeys, Brudzynski and Kim^59^ determined that the MIC value of honeys decreased by 50% in the first 3–6 months of storage, while the further decrease in antibacterial activity took place gradually against *Bacillus subtilis* and *Escherichia coli* bacteria. In our series of experiments, after one year, a 50–60% decrease in activity was observed in terms of the MIC values for each type of honey for all tested bacterial strains. The MIC values of the most active linden and sunflower honeys were 12.5% vs. 32.5% against the most resistant *P. aeruginosa* bacterium in 2022 vs. 2021, respectively.

Farkasovska et al.^61^ studied acacia, linden, rapeseed, sunflower and multifloral honey from Slovakia and found that in most honey samples H_2_O_2_ content correlated with their antibacterial activity, expressed as MIC. Similarly to our findings, sunflower and linden honeys exhibited the strongest antibacterial effect. While the H_2_O_2_ content of sunflower honeys showed correlation with their MIC values; linden honeys were exceptional in terms of antibacterial activity, which showed weak or no correlation with H_2_O_2_ content, depending on the bacterial strains. The outstanding inhibitory effect of linden honey has already been described in several studies, and factors other than H_2_O_2_ content were suggested as potential inhibitors of bacteria^53,61,65–67^. Few data are available regarding the level of antibacterial activity of sunflower honey, in this case the variety and geographical origin of the floral source may be an important influencing factor. Of the three Serbian honey samples studied by Đurović et al.^68^, sunflower honey had the second strongest antibacterial effect, which was higher than that of acacia honey, but lower than that of forest-meadow honey, with a MIC value of 12.5% against *Staphylococcus aureus* or *Serratia marcescens* bacteria. We also obtained similar results, the MIC value for *P. aeruginosa* was 12.5%, while for the other bacteria it was 10%. In accordance with the data published in our previous study, all Hungarian varieties of honey had an antibiofilm and bacteriostatic effect, but black locust honey and goldenrod honey showed less relevant inhibition^53,54^.

Similar to what was observed in our previous series of membrane degradation experiments^53^, the amount of DNA released from bacterial cells was more significant for Gram-positive *S. pneumoniae* than for Gram-negative *P. aeruginosa* bacteria after linden honey treatment. The studies of Otmani et al.^69^ also support the fact that Gram-positive bacteria are less resistant to honey treatments due to their structure. However, several studies have been published in which Gram-negative bacteria proved to be more sensitive^68,70^, including one of our previous studies^54^, which supports this, where we used bacterial strains that cause wound infection. Although the outer membrane of these bacteria may provide greater protection, the destruction of the lipopolysaccharide layer increases the permeability of the outer membrane, which can greatly contribute to release of the genetic material of the bacterial cell^71^. These different results show that the structure of the bacteria may play a smaller role in the development of resistance, rather the specific defense mechanisms of each strain are decisive. In our series of tests, we were the first to explore which concentration of linden honey is required for the degradation of the bacterial membrane, depending on the storage time. To date no other study has examined the effect of varietal honeys on the bacterial membrane as a function of storage time.

## Conclusions

Honey has a strong antibacterial effect, however, in order for it to be an additional therapy to an antibiotic course, several aspects must be taken into account. Our study highlights the importance of the botanical origin of honey, as some types of honey can exert a more significant inhibitory effect, and it is also relevant that the treatment should be applied against the bacterial strains that are the most sensitive to the given honey type. In addition, our research also answered the question of how much the age of honey can influence its antibacterial effect, and to what extent the antibiofilm activity decreases depending on the storage time. Our research group was the first to investigate the antibiofilm effect of honey samples stored for different time periods, as well as one of the mechanisms behind this, bacterial membrane degradation and its kinetics. Based on our results, honeys suitable for medicinal purposes should be used as fresh as possible during the various treatments, since in this case they exert the most significant antibacterial activity.

## Data Availability

The datasets generated during and/or analysed during the current study are available from the corresponding author on reasonable request.
